# High-Mobility Topological Semimetals as Novel Materials for Huge Magnetoresistance Effect and New Type of Quantum Hall Effect

**DOI:** 10.3390/ma16247579

**Published:** 2023-12-09

**Authors:** Roberto Zivieri, Stefano Lumetti, Jérémy Létang

**Affiliations:** 1 Consorzio Futuro in Ricerca (CFR), 44122 Ferrara, Italy; 2 Silicon Austria Labs, 9524 Villach, Austria; stefano.lumetti@silicon-austria.com (S.L.); jeremy.letang@silicon-austria.com (J.L.)

**Keywords:** topological semimetals, band structure, carrier mobility, magnetotransport properties, magnetoresistance effect, 3D quantum Hall effect, magnetoresistance sensors, high-speed devices, optoelectronic devices, photodetector devices

## Abstract

The quantitative description of electrical and magnetotransport properties of solid-state materials has been a remarkable challenge in materials science over recent decades. Recently, the discovery of a novel class of materials—the topological semimetals—has led to a growing interest in the full understanding of their magnetotransport properties. In this review, the strong interplay among topology, band structure, and carrier mobility in recently discovered high carrier mobility topological semimetals is discussed and their effect on their magnetotransport properties is outlined. Their large magnetoresistance effect, especially in the Hall transverse configuration, and a new version of a three-dimensional quantum Hall effect observed in high-mobility Weyl and Dirac semimetals are reviewed. The possibility of designing novel quantum sensors and devices based on solid-state semimetals is also examined.

## 1. Introduction

Since the beginning of this century, the investigation of the electrical transport properties of solid-state materials [1,2,3] has been a rapidly evolving field that has received a huge boost, especially after the discovery of graphene [4,5] and of its peculiar electrical transport and structural properties exhibited in different geometries (see, e.g., [6,7,8,9,10,11,12,13]) as well as of materials displaying topologically protected electronic states.

Recently discovered and widely studied classes of topological materials are the topological insulators, i.e., topologically-protected materials characterized by a non-zero surface electrical conductivity but preserving insulating properties in the bulk [14,15,16], and triple-point topological metals [17]. The concept of topological protection (and the interplay between topology and physics) has been extended to other types of materials featuring topological properties, with special regard to the novel class of topological semimetals, i.e., gapless semiconductors exhibiting linear dispersion bands in correspondence of the zero-gap points. Topological semimetals include binary or ternary compounds [18,19,20,21,22,23,24] characterized by specific surface states [25,26]. In this respect, semimetals, such as pentavalent elements arsenic, antimony and bismuth, and graphite form of carbon [2,3], have been known for a long time in solid-state physics and materials science together with their conduction features. Nonetheless, no topological properties have been associated with these elements.

In recent years, two additional classes of materials exhibiting topological properties similar to those of topological semimetals and insulators have been investigated: topological photonic crystals [27] and topological phononic crystals [28], which enabled to extend topology to classical wave systems as well. Topological photonics could enable the design of novel devices and applications, like topological quantum computation and the topological laser. By realizing artificially nanostructured units, viz. metamaterials, it is possible to create various topological states, by analogy to what occurs for quantum topological systems in condensed matter physics. Moreover, the recent introduction of topology and geometric phases in phononic crystals, represented in the form of classical acoustic and mechanical systems, has also allowed for the discovery of new physical and topological phenomena.

A new class of three-dimensional (3D) topological semimetals, the so-called Weyl semimetals, is characterized by non-degenerate bands touching at isolated points in the Brillouin zone (BZ). The experimental discovery of TaAs [29] was complemented by the observation of Fermi arcs [30,31,32] and the evolution of Fermi surface in the transition metal pnictide family [33], by a ternary type-II Weyl semimetal [34], by a hybrid Weyl semimetal [35], by the antiferromagnetic Mn${}_{3}$Ge and Mn${}_{3}$Sn [36] and by the magnetic Co${}_{3}$Sn${}_{2}$S${}_{2}$ [37], by the Dirac cone tilt in type-I and type-II Weyl semimetals [38], by the observation of the three-component fermions in MoP [39], by the electron-phonon interaction in $T_{d}$-WTe${}_{2}$ [40] and by an arc-tunable Weyl metallic state in Mo${}_{x}$W${}_{1-x}$Te${}_{2}$ [41]. They encompass binary and ternary compounds and are classified as type-I and type-II Weyl semimetals [22,23,42,43,44]. All of them exhibit massless excitations, referred to as Weyl fermions [45], characterized by divergent Berry curvatures around the band crossing points. The topological charges are represented by Weyl points (or nodes) in the momentum space hosting monopoles. In turn, the flux of the Berry curvature [21] flows from one monopole to the other defining the unique topological properties of a topological semimetal, which are strictly connected to its band structure [46,47]. An interesting physical consequence of the above-mentioned topological properties is that Weyl nodes lead to peculiar topologically protected surface states, named topological Fermi arcs, which are capable of connecting bulk Weyl points to opposite chiralities [22,30,48,49,50,51]. Fang et al. [52] also proved that multi-Weyl topological semimetals are stabilized by point group symmetry and can be regarded as new 3D topological semimetals.

The counterpart of Weyl semimetals, characterized by doubly-degenerate bands, is represented by 3D Dirac semimetals [42,53,54,55,56,57,58], which feature topological states with massless excitations, the so-called Dirac fermions [59,60]. The bands of the Dirac semimetals cross at the Dirac points on the Fermi surface, each being fourfold degenerate and resulting from the merging of a pair of Weyl points with opposite chiralities and thus characterized by a vanishing chiral charge or non-trivial Chern number [53,61,62,63].

Another recently discovered class of topological semimetals is that of nodal-line semimetals [64,65,66], in which the intersection of the conduction and the valence bands is a circular line [22,67] and which also exhibit peculiar magneto-optical properties [68]. This class of semimetals is characterized by type-I and type-II nodal lines as well [69,70,71]. Recently, attention has been given to the understanding and calculation of semimetal topological invariants [72,73], as was performed for the other classes of topological materials.

The theoretical and numerical approaches as well as the solid-state computational methods developed to determine the band structure of other classes of materials were recently applied to topological semimetals. Examples are the nearly free electron model, the k·p perturbation theory [1,74], the tight-binding (TB) model [75], and the muffin-tin approximation (enabling to determine the energy bands of *d*-type transition metal atoms and insulators), the linear combination of atomic orbitals (LCAO) technique [76], the augmented-plane-wave (APW) method [77] (used to calculate the band energy of transition metals) and the ab initio total energy calculation using a plane-wave basis set [78,79]. The most frequently employed computational methodology, owing to its generality and simplicity, is the density functional theory (DFT) [80,81,82,83] together with its variations such as, e.g., the projected augmented-wave (PAPW) [84], the linearized augmented-plane-wave (LAPW) [85,86] (applied to periodic materials) and the full-potential linearized augmented-plane-wave (FLAPW) (applied to any type of crystalline materials) [87] methods. These computational techniques can be generally used to characterize the band structure of solids and to understand how this affects charge carrier velocity and mobility.

The application of the above-mentioned computational methods to topological semimetals [18,22,88,89] enabled the understanding of the main differences between the energy band structure of semimetals, graphene, and III–V semiconductors. Semimetals exhibit a small overlap between the bottom of the conduction band and the top of the valence band with electrical transport properties determined by two types of carriers, electrons and holes [22]. They can be regarded as metals but, at the same time, they inherit the transport properties of semiconductors. They are 3D phases of matter with gapless electronic excitations protected by symmetry and topology. Nearly ideal Dirac semimetals have been modeled by Tang et al. [90]. A Dirac semimetal phase with quantum transport origin has been observed in Cd${}_{3}$As${}_{2}$ [91], while a nontrivial Berry phase characterizes the layered material PdTe${}_{2}$ [92] and type-II Dirac semimetals include the YPd${}_{2}$Sn class [93]. On the other hand, examples of typical Weyl semimetals are single-crystal transition-metal monophosphides [94,95], magnetic compounds like the canted antiferromagnet YbMnBi${}_{2}$ [96], and WTe${}_{2}$, whose centrosymmetric phase [97] coexists with topological insulator features [98].

A crucial role in determining the electrical transport properties is played by carrier mobility $\mu_{e,h}$—where *e* (*h*) denotes electrons (holes)—which describes the velocity of charge carriers in a metal, semimetal or semiconductor under the effect of an applied electric field. It is directly proportional to the relaxation time and inversely proportional to the effective mass, namely $\mu_{e,h}=\frac{e\tau }{m_{e,h}^{*}}$, τ being the relaxation time (i.e., the characteristic time between two successive scattering events) and $m_{e}^{*}$ ($m_{h}^{*}$) the electron (hole) effective mass. The mobility achieves its maximum value at low temperatures because of electron back-scattering protection (as occurs, e.g., in TaAs [99]), while it diminishes with increasing temperature [69,94,100,101] due to the enhanced scattering of charge carriers with longitudinal acoustic and optical phonons occurring at intermediate and high temperatures and attains a minimum at room temperature, similar to what occurs in semiconductors, graphene, and graphite [74,102,103,104].

The purpose of this review, unlike recent excellent reviews generally reporting on the state-of-the-art of topological semimetals (see, e.g., [22,88,89]), is to focus on the relationship between the electrical transport properties (like the high charge carrier mobility) of topological semimetals and their magnetotransport properties, including the large magnetoresistance (MR) and the novel 3D quantum Hall effect. This is achieved by providing a simple description of the interplay between the topological properties and the underlying physics characterizing these new classes of solid-state materials.

**Figure 1:**
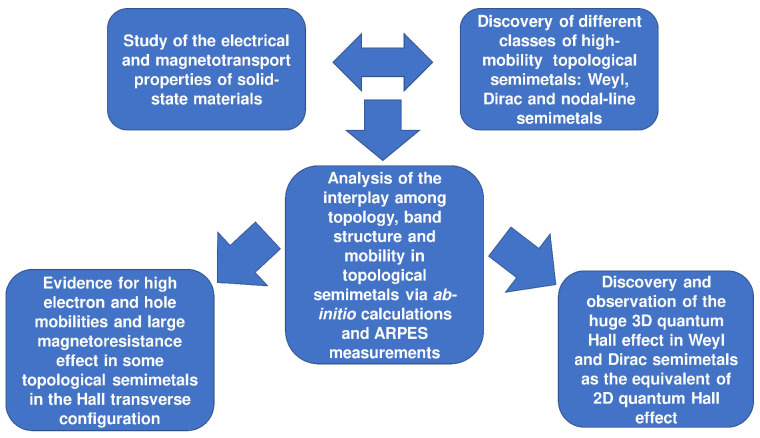
Graphical sketch illustrating schematically the motivation, the methodology, and the main magnetotransport properties results obtained for the most representative semimetals.

The study of the magnetotransport properties combined with the one of the electrical properties in well-known solid-state materials has boosted the discovery of different classes of topological semimetals characterized by different physical and topological properties. The motivation, the theoretical methods, the experimental observations, and the main findings obtained in terms of magnetotransport properties of high-mobility topological semimetals are shown in Figure 1.

## 2. Band Structure and Electrical Transport Properties in High-Mobility Semimetals

The strict relationship among band structure, electronic properties, and topology has a direct effect on the electrical properties of semimetals via the strong influence of the band curvature on carrier mobility and, in turn, on the electrical conductivity σ (due to the proportionality between σ and carrier mobility). In analogy with semiconductors [74,104], it is reasonable to suppose that the inverse of the electron effective mass $m_{e}^{*}$ and of the hole effective mass $m_{h}^{*}$ is proportional to the conduction and valence band curvature, respectively. At any temperature, the more the band curvature is accentuated, the larger are electron and hole mobilities $\mu_{e}$ and $\mu_{h}$, and the semimetal electrical conductivity tends to increase (also depending on carrier concentrations).

In this respect, it is widely known that carrier mobility in several semimetals often exhibits high values [69,94,105]. Indeed, carrier mobilities in semimetals are much higher than those of electrons in metals and of electrons and holes in intrinsic and doped semiconductors and graphene. For “high-mobility” semimetals, it is understood here that the charge carrier mobility overcomes the 10${}^{4}$ cm${}^{2}$· V${}^{-1}$· s${}^{-1}$ threshold at temperatures close to T=0 K and is not lower than 10${}^{2}$ cm${}^{2}$· V${}^{-1}$· s${}^{-1}$ close to room temperature. Most of the topological semimetals feature this property and their high carrier mobility leads to the observation of high electrical conductivity and huge MR even at low external magnetic fields [106,107]. MR is defined as the change in electrical resistance due to an external magnetic field applied either perpendicular (Hall configuration) or parallel or forming an arbitrary angle with the direction of the electric current flowing in the material under study. At the fixed external magnetic field, the MR strongly depends on carrier mobility, and high-mobility semimetals exhibit huge and non-saturating MR even at low fields and at temperatures close to room temperature [94,108,109,110]. The large MR, especially observed in the Hall configuration, is an appealing feature to be exploited for the design of semimetal-based magnetoresistive sensors, which could offer advantages in terms of sensitivity and precision if compared with other types of solid-state sensors like graphene-based sensors.

### 2.1. Relation between Topology and Physics

The new classes of Dirac, Weyl, and nodal-line semimetals originate from applications of the quantum relativistic theory to fermionic systems. Indeed, they can be regarded as solutions to Dirac and Weyl equations that are represented by Dirac and Weyl fermions [32,111].

**Figure 2:**
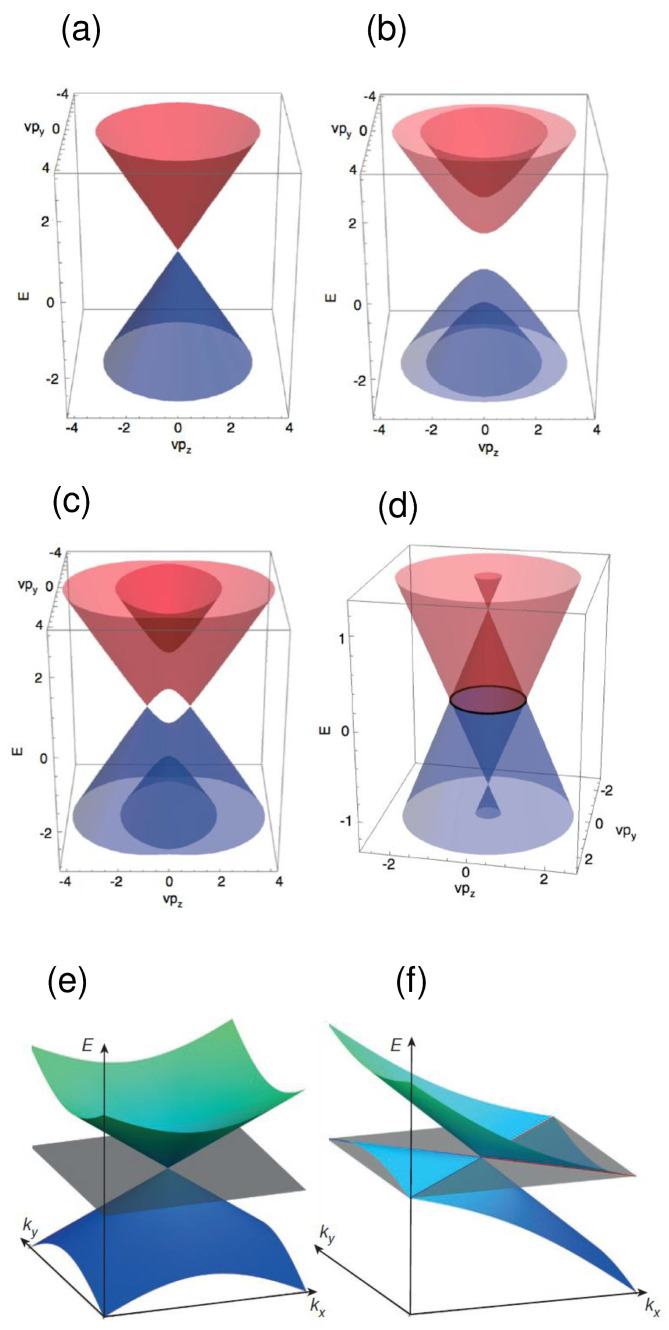
3D representation of band diagrams: (**a**) Dirac semimetals; (**b**) Semiconductors; (**c**) Weyl semimetals; (**d**) Nodal-line semimetals (adapted with permission from [22], Copyright 2018 American Physical Society); (**e**) Type-I Weyl point semimetal exhibiting a point-like Fermi surface. The Weyl point corresponds to the intersection point of the valence and conduction bands at the Fermi surface; (**f**) Type-II Weyl point semimetal corresponding to the contact point of electron and hole pockets (adapted with permission from [23], Copyright 2015 Springer Nature).

Figure 2a shows that the conduction and valence bands of a Dirac semimetal which match at a point lying on the Fermi surface at the wave vector k=0, referred to as Dirac point. This feature differs from the energy gap of a semiconductor, as illustrated in Figure 2b. The 3D band structure of a Weyl semimetal displayed in Figure 2c is characterized by a symmetric pair of isolated point nodes, the Weyl points, i.e., the matching points between electron and hole pockets on the Fermi surface. The Weyl points become a line in nodal-line semimetals (Figure 2d). Figure 2e shows the Weyl point for a type-I Weyl semimetal, while Figure 2f illustrates the Weyl point typical of a type-II Weyl semimetal.

Type-I and type-II Weyl semimetals [71,112,113,114,115,116,117,118,119,120] can be thought of as 3D phases of matter having electronic properties analogous to those of graphene. They exhibit gapless electronic excitations, the so-called Weyl fermions, which are solutions to the Weyl equation in odd dimensions. Weyl fermions are protected not only by symmetry, but also by topology, and can also possess relativistic properties in the presence of a magnetic field [121]. On the other hand, Dirac semimetals feature another type of gapless electronic excitations, the Dirac fermions, solutions to the Dirac equation.

**Figure 3:**
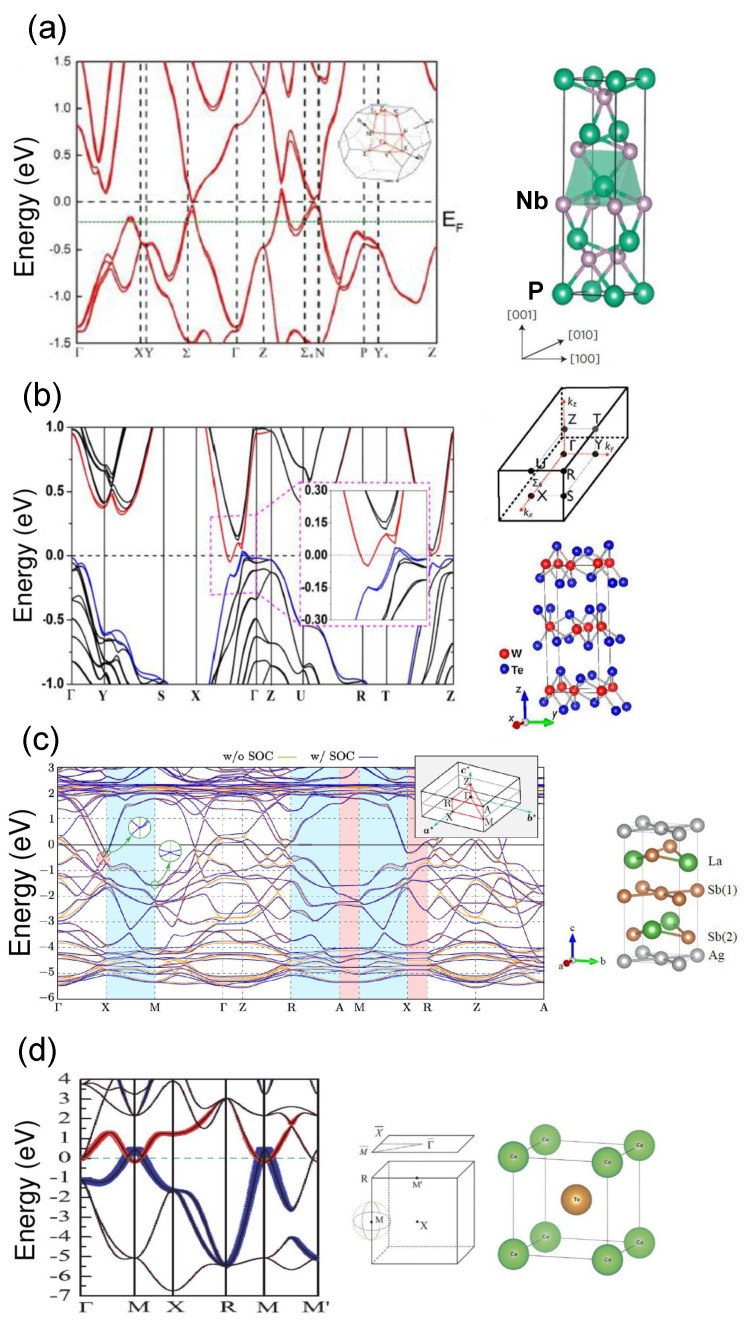
(**a**) Calculated band structure of type-I Weyl semimetal NbP together with its 3D BZ. The Fermi level intersects the valence band (adapted from [122]). The NbP tetragonal Bravais lattice is shown (adapted with permission from [94], Copyright 2015 Springer Nature); (**b**) Calculated band structure of WTe${}_{2}$ type-II Weyl semimetal. Red curves: contribution from W-5d orbital. Blue curves: contribution from Te-(i)−5p orbital where *i* indicates that the calculation was performed with Te atoms shrunk inside the sandwich layer. Black curves: Total contribution. Dashed violet rectangle: magnification of the band structure in the region including the minimum of the conduction band and the maximum of the valence band along the X-Γ direction of the 3D BZ. The 3D BZ and the distorted orthorombic Bravais lattice with octahedral coordination are displayed (each Tungsten layer is sandwiched between two Tellurium layers with strong ionic bonds) (adapted with permission from [123], Copyright 2015 IOP Publishing, and adapted from [124]); (**c**) Calculated band structure of LaAgSb${}_{2}$ Dirac semimetal in the absence of spin-orbit coupling (SOC) (orange lines) and in the presence of SOC (blue lines). Two circular insets, indicated by green arrows, represent the bulk Dirac cones appearing at the X and M points of the 3D BZ. The 3D BZ together with the tetragonal lattice are depicted (adapted from [125]); (**d**) Band structure without spin-orbit coupling of the CaTe mixed nodal-line and Dirac semimetal in CsCl-type phase. The valence and conduction bands get contributions from the $5p_{z}$ state of Te (blue line) and from the $3d_{z}^{2}$ state of Ca (red line). The weights of Te-$5p_{z}$ and of Ca-$3d_{z}^{2}$ are proportional to the width of blue and red curves, respectively. The 3D BZ projected on the plane and the bcc lattice are also shown (adapted from [67]).

Calculated band structures of some representative semimetals are displayed in Figure 3. Figure 3a shows the 3D bands of NbP, a type-I Weyl semimetal [94,122], determined by DFT. The intersection of the Fermi level with the valence band has an effect on carrier mobility and electrical conductivity, which noticeably increases due to a larger contribution from holes populating the valence band if compared with the contribution resulting from electrons in the conduction band. In turn, the MR also takes on extremely large values, even at room temperature, but only for rather high transverse magnetic fields. Figure 3b [123,124] displays the bands of the type-II Weyl semimetal WTe${}_{2}$. The calculated band structure of the Dirac semimetal LaAgSb${}_{2}$ is shown in Figure 3c [125], while Figure 3d illustrates the band structure of a CaTe mixed nodal-line and Dirac semimetal [67].

Experimentally, the band structure of semimetals can be reconstructed by means of angle-resolved photoemission spectroscopy (ARPES), a technique enabling to probe the surface states of solid-state systems [94,126]. On the other hand, powder X-ray diffraction and single-crystal X-ray diffraction are the most widely used experimental techniques to investigate the structural and lattice properties of high-mobility semimetals [94].

### 2.2. Electrical Transport Properties: Carrier Mobility and Electrical Conductivity

The calculation of the band structure of high-mobility semimetals is pivotal for the full understanding of their electrical properties, particularly their electron and hole mobility, their corresponding electrical conductivities, and their dependence on temperature *T*, analogously to what happens for intrinsic as well as doped semiconductors [127].

It is reasonable to suppose that the topological semimetal carrier mobility is inversely proportional to the carrier effective mass, i.e., $\mu_{e}\left(\mu_{h}\right)\propto 1/m_{e}\left(1/m_{h}\right)$, which, in turn, depends on the curvature of the bands, namely $1/m_{e}\propto \partial^{2}E_{c}/dk^{2}$ ($1/m_{h}\propto \partial^{2}E_{v}/dk^{2}$), $E_{c}$ ($E_{v}$) being the conduction (valence) band energy [104]. The effective mass of electrons is generally much lower than that of holes, leading to a lower mobility of the latter. However, due to the peculiar band structure of semimetals (in which the Fermi level intersects the valence band), holes also play a key role in the electrical transport properties and give an appreciable contribution to the total electrical conductivity. The most investigated topological semimetals so far have been the type-I Weyl semimetals due to their peculiar transport properties.

**Figure 4:**
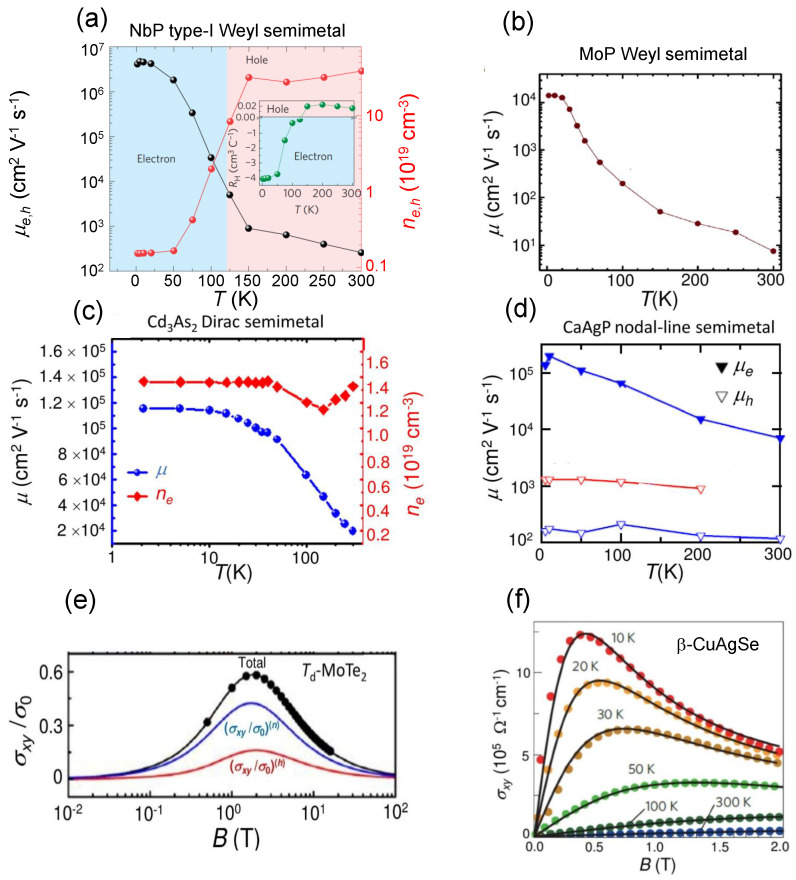
Measured temperature-dependent mobility, carrier density and electrical conductivity of representative topological semimetals. The mobility and the carrier density were extracted from Hall resistivity and Hall coefficient measurements. (**a**) Black line with circles: NbP type-I Weyl semimetal electron and hole mobility vs. *T* for 0<T≤300 K. Red lines with circles: carrier density vs. temperature for 0<T≤300 K. In the inset the dependence of the Hall coefficient on temperature *T* is also shown (adapted with permission from [94], Copyright 2015 Springer Nature); (**b**) Carrier mobility in the MoP Weyl semimetal vs. *T* (adapted from [100]); (**c**) Blue line with full circles: carrier mobility in the Cd${}_{3}$As${}_{2}$ Dirac semimetal as a function of temperature *T* for 0<T≤150 K extracted from a Hall measurement. Red line with diamonds: electron carrier density vs. *T* (adapted with permission from [101], Copyright 2015 American Chemical Society); (**d**) Red line with empty down-triangles: hole carrier mobility in the undoped CaAgP nodal-line semimetal vs. *T*. Blue line with empty down-triangles: hole carrier mobility in the Pd-doped CaAgP nodal-line semimetal vs. *T*. Blue line with full down-triangles: electron carrier mobility in the Pd-doped CaAgP nodal-line semimetal vs. *T* (adapted with permission from [69], Copyright 2020 American Physical Society); (**e**) Measured transverse electrical conductivity of $T_{d}$-MoTe${}_{2}$ type-II Weyl semimetal expressed in normalized units as a function of the applied magnetic field at T=2 K. Red curve: contribution of the holes to the electrical conductivity. Blue curve: contribution of the electrons to the electrical conductivity. Black curve: total normalized electrical conductivity (adapted with permission from [128], Copyright 2018 AIP Publishing); (**f**) Measured transverse electrical conductivity of CuAgSe phonon-glass electron crystal semimetal as a function of the applied magnetic field at different temperatures (adapted with permission from [129], Copyright 2013 Springer Nature).

In Figure 4 the measured electron and hole mobility dependence on temperature is displayed for four topological semimetals belonging to different classes. Even though the quantitative values may differ depending on the chosen semimetal for each class, the trends reported here can be considered general. At temperatures close to T=0 K, the NbP type-I Weyl semimetal exhibits the highest electron and hole mobility values. The Hall coefficient $R_{H}\left(T\right)$, calculated from the slope of the measured off-diagonal component of the resistivity $\rho_{xy}$, is negative in the range 0K<T<125K and positive for 125K<T≤300K (Figure 4a). Carrier density and carrier mobility can be extracted from the Hall coefficient $R_{H}\left(T\right)$ applying to Weyl semimetals a single-carrier band Drude model, namely $n_{e,h}\left(T\right)=1/\left(eR_{H}\left(T\right)\right)$ and considering the relation $\mu_{e,h}=R_{H}\left(T\right)/\rho_{xx}$, $\rho_{xx}$ being the diagonal component of the resistivity tensor and $R_{H}\left(T\right)$ a function of the electron (hole) density $n_{e}\left(T\right)\left(n_{h}\left(T\right)\right)$ [94] extracted from the slope of the off-diagonal component of the resistivity tensor $\rho_{xy}$ at high magnetic fields. The NbP compound can be regarded as a promising high-mobility material, because it incorporates the main transport features of the WTe${}_{2}$ type-II Weyl semimetal and the ones of the Cd${}_{3}$As${}_{2}$ Dirac semimetal. The NbP carrier mobility reaches a maximum value of about $5\;\times \;10^{6}$ cm${}^{2}\cdot$ V${}^{-1}\cdot$ s${}^{-1}$ at T=1.5 K allowing to consider NbP an ultra-high mobility semimetal. The MoP Weyl semimetal exhibits the lowest carrier mobility (Figure 4b) [100] if compared with the measured mobilities of carriers in other representative semimetals. This dissimilar behavior is related to the profound differences in the band curvatures of the various semimetals. Valence and conduction bands in NbP feature a higher curvature than those of other semimetals, which results in a dramatic increase in electron and hole mobility (especially at low temperatures). On the other hand, the smaller band curvature of MoP results in more than two orders of magnitude lower carrier mobility than that of NbP. It is also classified as a triple point topological metal, a metal with higher degenerate points exhibiting a high electrical conductivity [100]. Carrier mobility measurements for the Cd${}_{3}$As${}_{2}$ Dirac semimetal are shown in Figure 4c and are available only for 0<T≤150 K [101]. Hall resistivity measurements allow the separation of the electron and hole mobility in the CaAgP nodal-line semimetal shown in Figure 4d [69]. The Pd-doped CaAgP is a high-mobility semimetal and especially electron mobility attains a maximum at *T* close to 0 K with $\mu_{e}>10^{5}$ cm${}^{2}\cdot$ V${}^{-1}\cdot$ s${}^{-1}$. At room temperature the measured carrier mobility is higher in the CaAgP nodal-line semimetal (about $10^{4}$ cm${}^{2}\cdot$ V${}^{-1}\cdot$ s${}^{-1}$), although in this case the measurement was performed by doping the semimetal with Pd atoms. The undoped hole mobility in the CaAgP nodal-line Dirac semimetal is almost independent of *T*, is on the order of $10^{3}$ cm${}^{2}\cdot$ V${}^{-1}\cdot$ s${}^{-1}$ for the whole 0<T≤200 K range, and is higher with respect to the corresponding doped hole mobility.

The trend of carrier density ($n_{e,h}$) as a function of *T* is very different passing from a type-I Weyl semimetal to a Dirac semimetal. By comparing the NbP electron and hole density (red lines with circles in Figure 4a)) with the Cd${}_{3}$As${}_{2}$ electron density (red line with diamonds in Figure 4c), it can be observed that the former dramatically increases with *T* due to a strong contribution from holes at intermediate and high temperatures close to room temperature, while the latter is almost independent of *T* showing a dip at T≃130 K, and its value is one order of magnitude smaller than $n_{e,h}$ of NbP (which also includes the hole contribution).

Figure 4e,f show the behavior of the measured transverse electrical conductivity $\sigma_{xy}$ as a function of *B* for the $T_{d}$-MoTe${}_{2}$ type-II Weyl semimetal at T=2 K and for the β-CuAgSe phonon-glass semimetal [128,129]. For this latter semimetal, in the range of *B* considered, up to T=50 K, $\sigma_{xy}$ attains a maximum for both electrons and holes and then it decreases with *B* reproducing the trend given in Equation (3) (see next section). The maximum of $\sigma_{xy}$ shifts towards higher values of *B* tending to disappear for T>100 K in the range of *B* investigated, and decreases with increasing *T*. This trend confirms that, at fixed *B*, electron and hole conductivities diminish with increasing *T* due to the strong reduction in their respective mobilities, ultimately caused by the scattering with longitudinal acoustic and optical phonons and with lattice impurities. This behavior is accentuated the more the external magnetic field increases, especially in the range of small *B*.

## 3. High-Mobility Semimetals and Magnetotransport Properties

To determine the relationship between the electronic and the magnetotransport properties of all types of topological semimetals, it is crucial to quantify the MR effect, a physical phenomenon observed in different types of materials consisting in the change of electrical resistance due to the application of an external magnetic field. Several studies led to the discovery of different types of MR occurring in ferromagnetic, non-ferromagnetic metals and semiconductors, such as anisotropic MR (AMR), negative MR [130], tunnel MR (TMR) [131], giant MR (GMR) [132,133], colossal MR (CMR) [134] and extraordinary MR (EMR) [135]. Furthermore, other MR effects, such as positive MR [136] and Shubnikov-de-Haas (SdH) oscillations, have been observed in metals while geometrical MR has been detected in semiconductors [137]. As a natural continuation, great attention has been paid in recent years to exploring the MR in semimetals. It has been observed that MR is enhanced in some semimetals due to their structure and electrical properties, and is larger if compared with the one observed in the other classes of solid-state materials. This property makes semimetals appealing for information storage applications and for novel types of magnetic sensors.

Another relevant physical phenomenon recently discovered in some types of semimetals, which is also related to their electronic properties, is the 3D version of the quantum Hall effect [138] generally observed in two-dimensional (2D) systems.

Regarding the above two phenomena, it is important to give a brief illustration of the dependence on the frequency of the topological semimetals, and how this dependence affects magnetotransport properties. At sufficiently low temperatures and high magnetic fields, the free electrons in the conduction band of a semimetal behave like simple harmonic oscillators whose oscillation period changes proportionally to the magnitude of the external magnetic field. The more the magnetic field increases, the more energy levels pass through the Fermi energy and electrons populating them become free to flow as a current. This causes the material’s transport and thermodynamic properties to oscillate periodically, producing a measurable frequency oscillation in the topological semimetal’s electrical conductivity known as the SdH. The electronic frequency oscillation affects both the magnetoresistance at very low temperatures and the 3D quantum Hall effects. However, this correlation is more evident by studying either the quantum Hall resistance as a function of *B* or the quantum Hall conductance as a function of 1/B.

In the following subsections, these two effects (strictly dependent on the high-mobility properties of the topological semimetals) are reviewed and some applications thereof are discussed.

### 3.1. Magnetoresistance Effect: Theoretical Framework and Applications

The MR can be measured in different geometric configurations depending on the relative angle between the direction of the electric current *I* and the magnetic field B. In the case of semimetals, the most common MR measurement is performed in the Hall configuration, i.e., with B oriented out-of-plane and perpendicular to the direction of the current. The main source of MR in semimetals is related to the deviation of the charge carrier (both electrons and holes) trajectory due to the Lorentz force (Hall effect). In recent years, great attention has been devoted to the MR effect in high-mobility semimetals, and it has been found that topological semimetals exhibit an extremely large MR effect [108,109,139,140,141,142,143,144] as occurs for topological insulators [145]. Quantitatively, the MR can be expressed in percentage units as:

(1) $$\mathrm{MR}\left(\%\right)=\left(\frac{\rho (B)-\rho (0)}{\rho (0)}\right)\times 100\%,$$

where ρ(B) is the electrical resistivity at B≠0 T and ρ(0) is the resistivity at B=0 T, i.e., $\rho \left(0\right)=\rho_{0}$. For both electrons and holes (i=e,h), the field-dependent electrical resistivity ρ and electrical conductivity σ in matrix form at low and intermediate magnetic fields can be written as [146]:

(2) $$\rho =\rho_{0}\left(\begin{array}{cc} 1 & \mu_{i}B\\ -\mu_{i}B & 1 \end{array}\right),$$

(3) $$\sigma =\frac{\sigma_{0}}{1+{\left(\mu_{i}B\right)}^{2}}\left(\begin{array}{cc} 1 & -\mu_{i}B\\ \mu_{i}B & 1 \end{array}\right),$$

$\sigma_{0}=\sigma \left(0\right)$ being the electrical conductivity at B=0 T. Specifically, for fixed mobility $\mu_{i}$, at low *B* the off-diagonal component of the electrical conductivity tensor $\sigma_{xy}\propto \mu_{i}B$, namely it linearly increases with the external magnetic field, while at intermediate and high fields $\sigma_{xy}\propto 1/\left(\mu_{i}B\right)$, i.e., the electrical conductivity rapidly diminishes with increasing *B* (see Figure 4e,f).

In general, the MR is huge even for small external magnetic fields in high-mobility semimetals, especially at low temperatures at which the carrier mobility and the electrical conductivity are remarkable. Topological semimetals, both of the Dirac and Weyl types, show high MR due to a balanced hole-electron resonance effect resulting from their peculiar band structure; therefore, there is not only a contribution to MR from electrons but also from holes. As a consequence, the MR does not saturate at low and intermediate *B*, as occurs for semiconductors and graphene.

**Figure 5:**
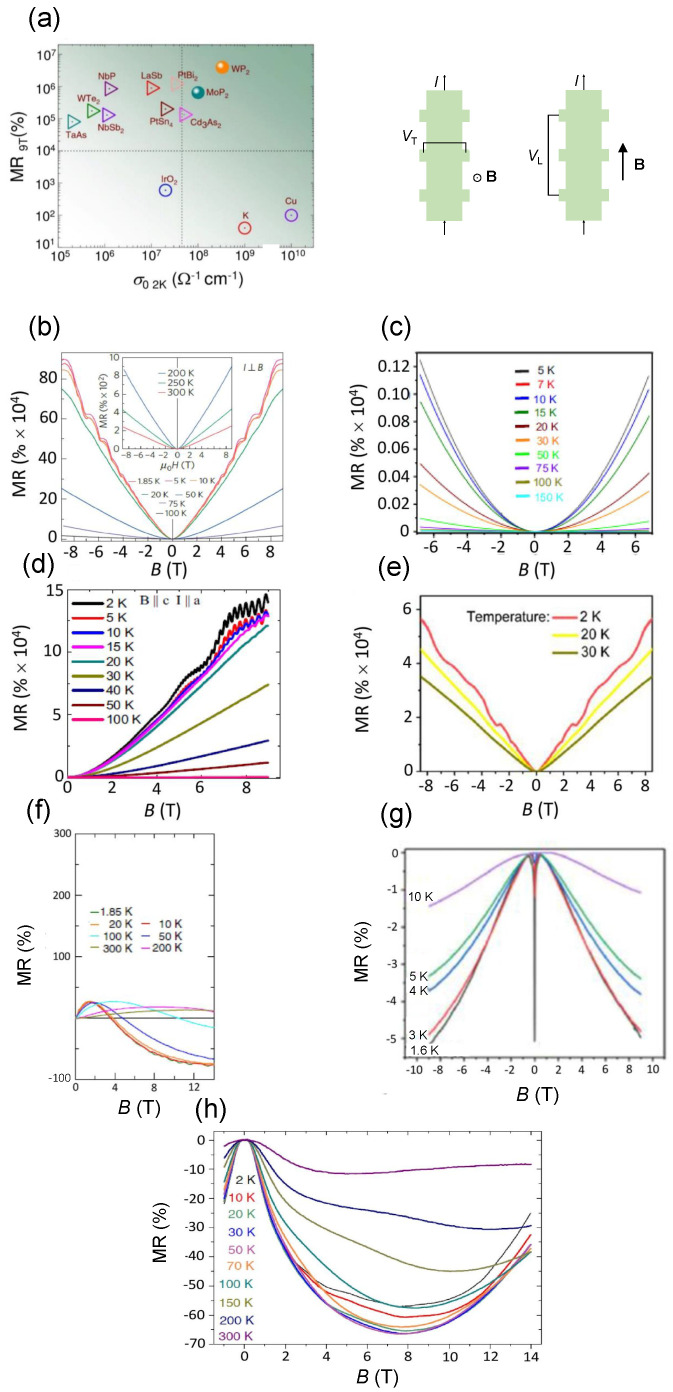
(**a**) Observed MR at *B* = 9 T and at T=2 K as a function of the electrical conductivity for representative topological semimetals in the Hall configuration. Triangles: Weyl semimetals and Dirac semimetals. Full circles: type-II Weyl semimetals WP${}_{2}$ and MoP${}_{2}$ semimetals. As a comparison, the MR of metals, K, and Cu, together with that of a metal oxide, IrO${}_{2}$, (hollow circles) are also depicted (adapted from [108]). MR (%) of some representative different types of semimetals measured in the Hall configuration (B⊥I) at different temperatures with B, the out-of-plane external magnetic field for: (**b**) An NbP type-I Weyl semimetal (adapted with permission from [94], Copyright 2015 Springer Nature); (**c**) A $T_{d}$-WTe${}_{2}$ type-II Weyl semimetal (adapted from [144]); (**d**) A ZrSiS Dirac semimetal with a and c two vectors along *x* and along *z*, respectively (adapted from [109]); (**e**) A ZrGeSe nodal-line semimetal (adapted with permission from [110], Copyright 2021 American Chemical Society). The geometries corresponding to the Hall MR configuration (B⊥I), with $V_{T}$ the transverse potential, and to the longitudinal MR configuration (B∥I), with $V_{L}$ the longitudinal potential, are also shown. MR (%) of some representative different types of semimetals measured in the longitudinal configuration (B∥I) at different temperatures with B, the in-plane external magnetic field for (**f**) A TaP type-I Weyl semimetal (adapted from [147]); (**g**) A TaAs${}_{2}$ type-II Weyl semimetal transitioning from the topological insulator state as soon as an external magnetic field is applied (adapted with permission from [126], Copyright IOP Publishing); (**h**) A Cd${}_{3}$As${}_{2}$ Dirac semimetal (adapted from [46]).

In Figure 5, the general trend of measured MR for some representative topological semimetals is displayed. Figure 5a graphically expresses the relationship between the measured MR (%) and electrical conductivity $\sigma_{0}$. This dependence is shown for MR (%) probed at T=2 K and at B=9 T [108] and for $\sigma_{0}$ measured at T=2 K. The highest MR(%) (almost $10^{7}$) is measured in WP${}_{2}$ (a type-II Weyl semimetal) at an electrical conductivity of about $10^{9}$$\Omega^{-1}$ cm${}^{-1}$ and a slightly lower value is exhibited by MoP${}_{2}$, another type-II Weyl semimetal. Lower but still considerable values of MR (%) (about $10^{5}-10^{6}$) are typical of the other shown Weyl and Dirac semimetals (triangles).

It is well-known that MR is proportional to resistivity but is inversely proportional to electrical conductivity, so the MR effect is significant in low-conductivity materials. However, looking at Figure 5a, type-II WP${}_{2}$ and MoP${}_{2}$ Weyl semimetals simultaneously exhibit large MR and rather high values of electrical conductivity larger than those of the Cd${}_{3}$As${}_{2}$ Dirac semimetal. Specifically, the electrical conductivity of WP${}_{2}$ is almost the same order of magnitude as the one of K and about one order of magnitude smaller than that of Cu. Therefore, type-II Weyl WP${}_{2}$ and MoP${}_{2}$ semimetals are materials with large electrical conductivity comparable to those of typical metals. This means that they can be regarded as materials very similar to metals, and with electrical transport properties not only determined by electrons as in metals but also by holes.

Figure 5b–e display MR measurements of typical high-mobility semimetals as a function of the applied external magnetic field at different temperatures in the Hall configuration, i.e., the out-of-plane external magnetic field B is perpendicular to the in-plane current (θ=90°). The two general observations for all the semimetals, which can be drawn looking at Figure 5b–e, are: (1) MR does not reach a saturation value but tends to increase with increasing *B* for low and intermediate *B* and (2) MR decreases with increasing *T* as in III–V semiconductors, but it is still huge at room temperature. Regarding (1), the contribution to MR due to holes in high-mobility semimetals is more relevant with respect to the one present in III–V semiconductors giving rise to a more accentuated electron-hole resonance effect which leads to a larger MR value in semimetals. Concerning (2), in high-mobility semimetals, carrier mobility decreases with increasing temperature due to the large scattering of electrons and holes with acoustic and optical phonons, especially at intermediate and high *T* close to room temperature, but the effect of scattering is partially masked by the appreciable contribution of holes to the mobility.

One notes that there are quantitative differences among the measured MR (%) values depending on the type of semimetal. In particular, the highest values at any temperature are exhibited by the NbP type-I Weyl semimetal. On the other hand, for the same magnetic field, the MR (%) for the representative Dirac semimetal is almost one order of magnitude less than the one exhibited by type-I Weyl semimetal, while the nodal-line semimetal has a MR (%) comparable to the one of Dirac semimetal. The reason for these discrepancies is attributed to the higher carrier mobility characterizing type-I Weyl semimetal, which leads to a more accentuated increase in the field-dependent resistivity with increasing *B* as expressed by Equation (2). Finally, it must be emphasized that the measured MR(%) of $T_{d}$-WTe${}_{2}$ type-II Weyl semimetal is much lower if compared to those of the other shown semimetals. However, there are type-II Weyl semimetals (e.g., WP${}_{2}$) exhibiting a MR (%) even higher than those of type-I semimetals, such as NbP (see Table 1 for a comparison). Therefore, there is also a strong dependence on the type-II Weyl semimetal examined.

MR is typically measured for other geometries ($0<\theta <90^{o}$) and in the so-called longitudinal configuration with B∥I. As shown in Figure 5f–h [112,126,147,148], MR as a function of *B* is negative for all the fields with the exception of the TaP type-I Weyl semimetal (Figure 5f) where it is negative above a given external magnetic field and for T≤100 K. When B is not orthogonal to the direction of the current (and, as a result, to the electric field E), the quantum mechanical conservation of the particle number does not hold anymore: this is the so-called Adler–Bell–Jackiw anomaly or chiral anomaly, which manifests itself in the presence of well-defined chirality appearing when the Fermi level is close to the Weyl nodes [147]. It can be proved that chiral anomaly in Weyl semimetals leads to negative MR vs. B with the absolute value of MR increasing as temperature decreases, and this effect is maximum when B∥E. In the presence of a chiral anomaly, the external magnetic field suppresses the phase coherence of the backscattered electron and hole waves and destroys the weak localization effect. The more B deviates from the collinear configuration with the current, the more the Lorentz force gives a positive contribution, which cancels the negative MR effect. Considering that the MR absolute value in this configuration is much lower with respect to that in the Hall configuration, the design of magnetoresistive sensors based on this effect could be useful but less exploitable if compared to that in the Hall geometry.

The mobilities of representative topological semimetals are summarized in Table 1 together with their MR(%) at low temperatures and at given values of the external magnetic fields [20]. Among the different types of semimetals, some of type-II Weyl semimetals are the ones exhibiting the highest MR(%) (see e.g., WP${}_{2}$), but type-I Weyl semimetals are also characterized by high mobility, especially at low temperatures (see e.g., NbP), and, as a result, also by a huge value of MR(%). Looking at Table 1, one notes that there is a strict relationship between the two physical quantities, as occurs for other types of high-mobility materials, such as graphene or some types of III–V semiconductors.

**Table 1:** Carrier mobilities, resistivities and MR obtained in the transverse Hall configuration for various applied magnetic fields of a few representative topological semimetals.

Semimetal	Mobility $\mu_{e,h}$ (cm${}^{2}\cdot$ V${}^{-1}\cdot$ s${}^{-1}$)	Resistivity $\rho_{0}$ (Ω· cm)	MR (%)
NbP [94]	$5\times 10^{6}$ (1.85 K)	$6.3\times 10^{-9}$ (2 K)	$8.5\times 10^{5}$ (1.85 K, 9 T)
${\mathrm{NbSb}}_{2}$ [149,150]	$2.1\times 10^{4}$ (5 K)	$8\times 10^{-9}$ (2 K)	$1.3\times 10^{5}$ (2 K, 9 T)
NbAs [151]	$3.5\times 10^{5}$ (2 K)	$1\times 10^{-8}$ (2 K)	$2.3\times 10^{5}$ (2 K, 9 T)
TaP [122,152,153]	$3\times 10^{5}$ (2 K)	$4\times 10^{-6}$ (2.5 K)	$2.31\times 10^{4}$ (5 K, 6 T)
TaAs [99]	$5\times 10^{5}$ (2 K)	$1\times 10^{-6}$ (1 K)	$5.4\times 10^{5}$ (10 K, 9 T)
${\mathrm{WP}}_{2}$ [108]	$4\times 10^{6}$ (2 K)	$3\times 10^{-9}$ (2 K)	$4.2\times 10^{6}$ (2 K, 9 T)
${\mathrm{WTe}}_{2}$ [142,154]	$5.0\times 10^{3}$ (1.6 K)	$1.9\times 10^{-6}$ (2 K)	$4.5\times 10^{5}$ (4.5 K, 14.7 T)
${\mathrm{PtSn}}_{4}$ [155,156]	$1\times 10^{5}$ (2 K)	$4\times 10^{-8}$ (2 K)	$5\times 10^{5}$ (1.8 K, 14 T)
${\mathrm{PtBi}}_{2}$ [157]	$3.1\times 10^{4}$ (2 K)	$2.4\times 10^{-8}$ (2 K)	$1.12\times 10^{7}$ (1.8 K, 33 T)
${\mathrm{Cd}}_{3}{\mathrm{As}}_{2}$ [140]	$9\times 10^{6}$ (5 K)	$2\times 10^{-5}$ (2 K)	$3.1\times 10^{3}$ (2 K, 9 T)
${\mathrm{MoP}}_{2}$ [108]	$4\times 10^{6}$ (2 K)	$1\times 10^{-8}$ (2 K)	$3.2\times 10^{5}$ (2 K, 9 T)
MoP [100]	$1\times 10^{4}$ (2 K)	$6\times 10^{-9}$ (2 K)	$3.2\times 10^{3}$ (2 K, 14 T)

### 3.2. Novel Type of Quantum Hall Effect in 3D Topological Semimetals

Very recently, a 3D quantum Hall effect has been reported in some types of high-mobility topological semimetals [138,158,159]. Indeed, topological semimetals can be seen as 3D topological states of matter. The 3D quantum Hall effect can be considered the equivalent in three dimensions [160] of the usual 2D quantum Hall effect [161,162]. The latter, also called the integer quantum Hall effect to distinguish it from the fractional quantum Hall effect, is the corresponding quantum version of the Hall effect. It consists of the quantization of the Hall conductance (or equivalently of the Hall resistance) in 2D (*x*-*y* plane) electron gas systems at low temperatures, (typically at liquid helium temperature T=4.2 K), in the presence of a strong external magnetic field (*B* several Tesla) applied perpendicularly to the plane of the charge carrier system. This effect results from the evolution of a 2D electron gas into Landau levels which deform at the edges and cut through the Fermi energy giving rise to 1D edge states protected by topology. The quantized Hall conductance due to electrons flowing through the edge states can be written as $G_{H}=I/V_{\mathrm{Hall}}$, where *I* is the electrical current, and $V_{\mathrm{Hall}}$ the Hall potential and can be expressed as $G_{H}^{2D}=\frac{n\;e^{2}}{h}$ (where n=1,2,… is a non-negative integer expressing the $G_{H}^{2D}$ quantization in terms of Landau levels lying below the Fermi energy $E_{F}$, *e* is the electron charge and *h* is the Planck constant). In 2D systems, $G_{H}^{2D}$ has the dimensions of $e^{2}/h$ (expressed in Siemens (S) with $S=\Omega^{-1}$), while in 3D systems $G_{H}^{3D}$ has the dimensions of $e^{2}/h$ over length (expressed in S/m).

The main observed feature is the exhibition of the quantum Hall conductance plateaus at $\frac{e^{2}}{h},\frac{2e^{2}}{h},\ldots$ either as a function of 1/B or of the carrier density. Equivalently, the quantum Hall resistance $|R_{\mathrm{xy}}|=\frac{h}{n\;e^{2}}$ is characterized by plateaus at $\frac{h}{e^{2}},\frac{h}{2e^{2}},\ldots$ as a function of *B* or of the electron density. This result looks surprising because quantum Hall conductance does not explicitly depend on carrier density. High-mobility materials like the family of TaAs Weyl semimetals and the Cd${}_{3}$As${}_{2}$ and Na${}_{3}$Bi Dirac semimetals satisfy the main requirement necessary to exhibit the 3D quantum Hall effect, which consists in the presence of Fermi arcs placed on the Fermi surface between Weyl nodes and formed by the topologically protected states. By exploiting the tunnel effect, the electrons can pass through the Fermi arcs placed at opposite surfaces via a “wormhole” tunneling supported by the Weyl nodes [138]. In addition, in order to have the 3D quantum Hall effect: (1) the Fermi arcs should also form closed Fermi loops, (2) bulk carriers must be depleted by tuning the Fermi energy to the Weyl nodes, and (3) there should be band anisotropy so that the Fermi arcs give rise to a 2D gas [138]. Due to time-reversal symmetry, a complete Fermi loop occurs on a single surface of the Cd${}_{3}$As${}_{2}$ and Na${}_{3}$Bi Dirac semimetals. In particular, the 3D quantum Hall effect based on Weyl orbits has been recently realized in the 3D Dirac Cd${}_{3}$As${}_{2}$ high-mobility semimetal in nanostructured form [159]. It is crucial to understand the behavior of Weyl orbits under an external magnetic field. There are two pairs of Weyl nodes of opposite chiralities acting like “wormholes” connected by Fermi arcs lying on two opposite surfaces in the $k_{x}$-$k_{z}$ plane, i.e., the top and the bottom Fermi arcs. Electron propagation occurs along the *z*-direction through the bulk chiral Landau levels in order to complete the cyclotron motion.

The quantized 3D Hall electrical conductivity for topological Weyl and Dirac semimetals can be calculated starting from the Kubo formula [138]:

(4) $$\sigma_{H}^{3D\;sm}=\frac{e^{2}\;\hbar }{iV_{\mathrm{eff}}}\sum_{\delta ,\delta^{\prime }\neq \delta }\frac{\langle \Psi_{\delta }\left|v_{x}\right|\Psi_{\delta^{\prime }}\rangle \langle \Psi_{\delta^{\prime }}\left|v_{z}\right|\Psi_{\delta }\rangle \left(f\left(E_{\delta^{\prime }}\right)-f\left(E_{\delta }\right)\right)}{\left(E_{\delta }-E_{\delta^{\prime }}\right)\left(E_{\delta }-E_{\delta^{\prime }}+i\Gamma \right)},$$

where sm= Weyl or Dirac, $\Psi_{\delta }$ is the eigenstate corresponding to the energy $E_{\delta }$ when the external magnetic field is applied along the *y* direction, $v_{\alpha }$ (with α=x,z) is the velocity operator, $f(E_{\delta })$ is the Fermi distribution, $V_{\mathrm{eff}}$ is the effective volume of the slab or the areas hosting the Fermi arcs and Γ expresses the level broadening. Γ→0 means that the disorder is weak and does not affect the width of plateaus. The sheet 2D quantum Hall electrical conductivity having dimensions of $e^{2}/h$ (S) is obtained as $\sigma_{H}^{S\;s}=\sigma_{H}^{3D\;s}L$, *L* being the slab thickness. From Equation (4), at zero temperature the quantum Hall electrical conductivity $\sigma_{H}=J_{x}/E_{z}$ (with the current density along *x* and the induced electric field along *z*) of the Weyl topological semimetal is [138]:

(5) σH3DWeyl=te2h4LlB4∑Eδ<EF,Eδ′>EFRe(v¯xδδ′v¯zδ′δ)(Eδ−Eδ′)2+Γ2,

with t=sgn(eB), $l_{B}=\sqrt{\hbar /|eB|}$, ${\bar{v}}_{x}^{\delta \delta^{\prime }}\delta_{k_{x}^{\prime },k_{x}}=\hbar \frac{l_{B}}{\sqrt{2}}\langle \Psi_{\delta }\left|v_{x}\right|\Psi_{\delta^{\prime }}\rangle$, ${\bar{v}}_{z}^{\delta^{\prime }\delta }\delta_{k_{x}^{\prime },k_{x}}=\hbar \frac{l_{B}}{it\sqrt{2}}\langle \Psi_{\delta^{\prime }}\left|v_{z}\right|\Psi_{\delta }\rangle$ and $\sigma_{H}^{S\;\mathrm{Weyl}}=\sigma_{H}^{3D\;\mathrm{Weyl}}L$.

The quantum Hall conductance of Fermi arc I at the upper slab surface (y=L/2) can now be calculated. The effective Hamiltonian of the Fermi arc in a 3D Weyl topological semimetal having the form of a slab of thickness *L* (see Figure 5) can be derived from the Hamiltonian of a 3D Weyl topological semimetal and parameterized in the form $H_{\mathrm{arc}}=D_{1}k_{w}^{2}+vk_{x}+\left(D_{2}-D_{1}\right)\left(k_{x}^{2}+k_{z}^{2}\right)$ for the y=L/2 surface with $v=A\sqrt{M^{2}-D_{1}^{2}}/M$, $D_{1}$, $D_{2}$, *A* and *M* being model parameters and $k_{w}$ the wave vector of the Weyl node. The quantum Hall conductance of arc I $G_{H}^{\mathrm{arc}}$ for a Weyl topological semimetal slab (for Γ→0) obtained from the 2D electron gas quantum Hall electrical conductivity $\sigma_{H}=ne^{2}/h$ (with n=1,2,… the number of Landau levels below the Fermi energy) is [138]:

(6) $$G_{H}^{\mathrm{arc}\;\mathrm{Weyl}}=\frac{e^{2}}{h}\mathrm{sgn}\left(R\right)\mathrm{sgn}\left(eB\right)*\frac{S_{I}/{\left(2\pi \right)}^{2}}{eB/h}+\frac{1}{2},$$

with *R* = $D_{2}-D_{1}$, $S_{I}=2k_{w}^{2}(1+v^{2}/4R^{2}k_{w}^{2}\arctan(2|R|k_{w}/\left|v|\right)-\left|v\right|k_{w}/\left|R\right|$ the area filled by arc I in the momentum space and ⌊…⌋ denoting rounding down. The sign of the quantum Hall conductance depends on the signs of *R* and of eB, and therefore ultimately depends on carrier and charge type as well as on the direction of the external magnetic field. Similar conclusions can be drawn by considering Fermi arc II at the lower slab surface (y=−L/2).

Analogously, the quantum Hall electrical conductivity of a Dirac semimetal slab can also be calculated from (4) at zero temperature. It turns out to be [138]:

(7) σH3D Dirac=e2h8πty′Vly′2∑kx′∑Eδ<EF,Eδ′>EFRe(v¯xδδ′v¯zδ′δ)(Eδ−Eδ′)2+Γ2,

with $t_{y^{\prime }}=\mathrm{sgn}\left(eB_{y^{\prime }}\right)$, $l_{y^{\prime }}=\sqrt{\hbar /|eB_{y^{\prime }}|}$ ($B=(B_{x^{\prime }},B_{y^{\prime }},0)$ being the uniform magnetic field applied in the $x^{\prime }$-$y^{\prime }$ plane), *V* volume of the slab, $k_{x}^{\prime }$ is the $x^{\prime }$ component of the wave vector obtained after a rotation of the *y* axis with both velocity operators referred to the rotated reference frame to the $y^{\prime }$ axis, ${\bar{v}}_{x}^{\delta \delta^{\prime }}\delta_{k_{x}^{{}^{\prime \prime }},k_{x}^{{}^{\prime }}}=\hbar \frac{l_{y^{\prime }}}{\sqrt{2}}\langle \Psi_{\delta }\left|v_{x}\right|\Psi_{\delta^{\prime }}\rangle$, ${\bar{v}}_{z}^{\delta^{\prime }\delta }\delta_{k_{x}^{{}^{\prime \prime }},k_{x}^{{}^{\prime }}}=\hbar \frac{l_{y^{\prime }}}{it_{y^{\prime }}\sqrt{2}}\langle \Psi_{\delta^{\prime }}\left|v_{z}\right|\Psi_{\delta }\rangle$.

**Figure 6:**
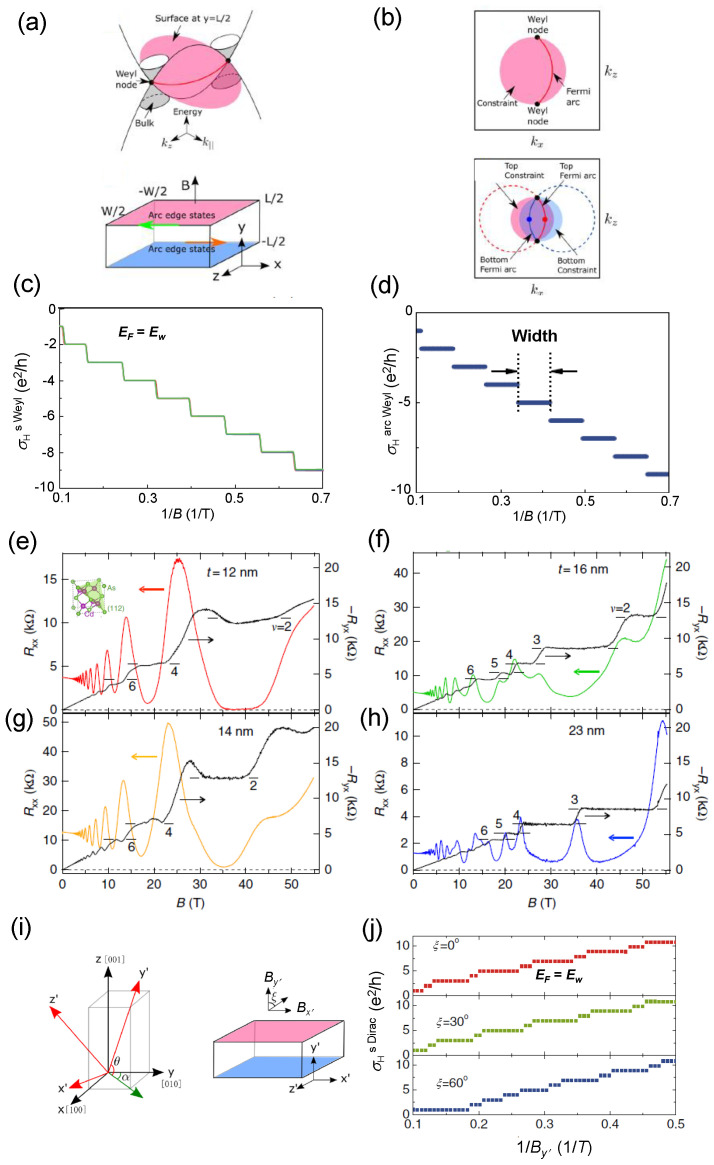
(**a**) Energy dispersion of a Weyl semimetal (e.g., TaAs family) and the slab of thickness *L* and width *W*. B is applied along the *y* direction. (**b**) Fermi arc at y=L/2 and $E_{F}=E_{w}$. The Fermi arcs can exist on the shadow area. The top (solid red line) and bottom (solid blue line) Fermi arcs at the y=L/2 and y=−L/2 surfaces of the slab at $E_{F}=E_{w}$ and the corresponding top and bottom constraints (pink shadow and cyan shadow areas); (**c**) The sheet quantum Hall electrical conductivity for the corresponding slab vs.1/B calculated by means of Equation (5) for $E_{F}=E_{w}$ where $E_{F}$ crosses the arc I. (**d**) Calculated quantum Hall conductance for arc I vs.1/B according to Equation (6) (here labeled with $\sigma_{H}^{\mathrm{arc}\;\mathrm{Weyl}}$) (adapted with permission from [138], Copyright 2017 American Physical Society); (**e**–**h**) Measured quantum Hall resistances in the topological Cd${}_{3}$As${}_{2}$ Dirac semimetal films vs.*B*. (**e**) Longitudinal resistance $R_{xx}$ (red line) and transverse Hall resistance $-R_{yx}$ (black line) for a *t* = 12 nm thin film. Inset: primary cubic cell of Cd${}_{3}$As${}_{2}$. (**f**) As in panel (**e**) with $R_{xx}$ (green line) and $-R_{yx}$ (black line) for a *t* = 14 nm thin film. (**g**) As in panel (**e**) with $R_{xx}$ (orange line) and $-R_{yx}$ (black line) but for a *t* = 16 nm thicker film with $R_{xx}$ (green line) and $-R_{yx}$ (black line). (**h**) As in panel (**e**) with $R_{xx}$ (blue line) and $-R_{yx}$ (black line) but for a *t* = 23 nm thicker film with $R_{xx}$ (blue line) and $-R_{yx}$ (black line). In panels (**e**–**h**) the numbers of the horizontal bars indicate the filling factor *f* and the left (right) arrows refer to $R_{xx}$ ($-R_{yx}$) (adapted from [158]); (**i**) The crystallographic directions ([100] along *x*, [010] along *y* and [001] along *z*) of Cd${}_{3}$As${}_{2}$ together with the coordinate system used for its modeling. $\left(\alpha ,\theta \right)=\left(-\pi /4,\arctan\sqrt{2}\right)$ correspond to the slab growth direction [112] and (α,θ)=(−π/4,0) to the [110] one. $y^{\prime }$ is the growth direction of the slab and the Hall conductance is defined in the $x^{\prime }$-$z^{\prime }$ plane. The angle ξ expresses the angle of rotation of B from the $y^{\prime }$ to the $x^{\prime }$ direction in the $x^{\prime }$-$y^{\prime }$-$z^{\prime }$ coordinate system with $B_{x^{\prime }}=B\sin\xi$ and $B_{y^{\prime }}=B\cos\xi$. (**j**) Calculated sheet Hall electrical conductivity of Cd${}_{3}$As${}_{2}$ at the Dirac node energy $E_{w}$ vs.$1/B_{y^{\prime }}$ according to Equation (7) with $E_{w}=E_{F}$ vs. 1/*B* for different orientations of the magnetic field (adapted with permission from [138], Copyright 2017 American Physical Society).

Figure 6 shows the 3D quantum Hall effect observed both in the TaAs topological Weyl semimetal family (Figure 6a–d) and in the Cd${}_{3}$As${}_{2}$ topological Dirac semimetal (Figure 6e–j). Figure 6a,b display the topological features of 3D TaAs in the form of a slab occurring in the presence of a 3D quantum Hall effect, including the energy dispersion and the topology of the Fermi arcs at $E_{F}=E_{w}$ with $E_{F}$ the Fermi energy and $E_{w}$ the Weyl node energy. In Figure 6c, the corresponding calculated sheet quantum Hall electrical conductivity vs. the inverse of the magnetic field shows the distinctive plateaus expression of the Landau level quantization of Hall electrical conductivity. An analogous behavior is depicted in Figure 6d for the quantum Hall elecrical conductance of Fermi arc I.

Figure 6e–h display the behaviors of the measured longitudinal resistance $R_{xx}$ and of the transverse Hall resistance $-R_{yx}$ on the *x*-*y* plane of Cd${}_{3}$As${}_{2}$ films of different thicknesses as a function of the magnetic field *B*. All films are characterized by the same carrier density $n=1\times 10^{18}$ cm${}^{-3}$. As soon as the external magnetic field is turned on, Shubnikov-de Haas (SdH) oscillations of the longitudinal resistance begin to appear exhibiting a decreasing frequency with increasing magnetic field magnitude. The vanishing of $R_{xx}$ occurs at B=35 T (Figure 6e) for a t=12 nm thin film. For all thicknesses $-R_{yx}$ (black lines) exhibit the typical plateaus marking the 3D quantum Hall effect.

Figure 6i illustrates the 3D quantum Hall modelization for a Cd${}_{3}$As${}_{2}$ slab (coordinate system $x^{\prime }$-$y^{\prime }$-$z^{\prime }$) grown along the [112] and [110] crystallographic directions and the corresponding calculated sheet Hall electrical conductivity as a function of $1/B_{y^{\prime }}$ for different orientations of the magnetic field marked by the angle ξ for $E_{F}=E_{w}$ (Figure 6j). Quantum Hall electric conductivity plateaus appear for any orientation of B and their widths are of different sizes for any χ investigated.

By observing the trend of the transverse Hall resistance $-R_{yx}$, the quantum Hall states begin to appear analogously to what occurs for the calculated Hall electrical conductivity. The quantized values can be expressed in the form $\frac{1}{R_{yx}}=-\nu \frac{\;e^{2}}{h}$, with ν=sn an integer number depending in this case not only on n=1,2,⋯ but also on the degeneracy factor *s*. One notes that the integer quantum Hall states clearly emerge for ν=2 for all the thicknesses considered. Moreover, the degeneracy factor drastically changes from *s* = 2 to *s* = 1 for the thickness passing from *t* = 14 nm to *t* = 16 nm. This change is due to spin splitting and is not related to other types of degeneracies, such as the lifting of the valley or surface states. Interestingly, by performing the Fourier transform of the SdH oscillations the area $A_{F}$ of the Fermi surface can be calculated via the Onsager relation as $A_{F}=\left(4\pi^{2}e/h\right)\;B_{F,1}$ where $B_{F,1}$ is the primary oscillation frequency. For example, for the *t* = 12 nm film $A_{F}=3.3\times 10^{-3}{Å}^{-2}$.

## 4. Conclusions and Outlook

In this review article, the magnetotransport properties of topological semimetals, prototypes of high-mobility materials, have been reviewed based on the recent theoretical and experimental work conducted during the last two decades on this novel class of materials. The relationship among carrier mobility, electrical conductivity, and magnetotransport shows that high-mobility topological semimetals exhibit huge MR, especially in the Hall configuration, even at low external magnetic fields, and could be excellent candidates to design MR-based high-precision magnetic quantum sensors. The high mobility is also at the basis of another recently discovered phenomenon, the 3D quantum Hall effect, in some types of topological Weyl and Dirac semimetals, which can be considered the 3D version of the widely known 2D quantum Hall effect. These novel investigations could boost the research trends towards a full understanding of the subtle physics that governs 3D topological systems and could extend the search for device applications related to 3D systems.

The main interest in the study of this class of materials is their strong response to an external magnetic field if compared to other classes of solid-state materials such as, e.g., semiconductors, due to their peculiar electronic structure topology around the Fermi surface and to the essential role of Fermi surface topology itself. These unique topological properties are advantageous. Indeed, they result in the enhancement of the effect of B on charge carriers, leading to a large MR effect just at low-intensity fields and temperatures close to room temperature and to its tendency to not saturate even at high B together with the exhibition of a relevant quantum Hall effect. Moreover, there is also an amplification of the effects of an external electric field resulting in a giant transverse and longitudinal magneto-thermoelectric (MTE) effect to be used for novel thermoelectric quantum materials. On the other hand, there are also disadvantages related to the fabrication process implying the formation of vacancies resulting in a lower charge carrier mobility, as shown for Mg${}_{3}$Bi${}_{2}$ nodal-line semimetal and, thus, limiting the MTE effect and, more generally, the magnetotransport properties [163].

The study of the effect of the electrical and magnetotransport properties of a metal on those of topological semimetals would be a novel research direction worth further investigation. This goal could be accomplished by simulating metal/semimetal heterojunctions (analogously to the cases of modulation-doped heterostructures [164] and to metal/graphene heterojunctions [165]), by using a typical metal having high electrical conductivity with either Dirac or Weyl topological semimetals and by calculating a probable increase in the mobilities of electrons and holes in the semimetal. Indeed, even though the electron mobility of a typical metal (e.g., Au) is smaller than the corresponding one of a topological semimetal, the electrical conductivity is, generally, at least one to two orders of magnitude larger. A huge enhancement of MR with respect to the case of the single semimetal layer should also be expected even at low fields and temperatures close to room temperature due to the consistent contribution of holes in the semimetal layer to electrical and magnetotransport properties. A confirmation of this behavior could be obtained by measuring: (1) the variation of the mobility $\mu_{e,h}$ experienced by the semimetal due to the generation of contact resistance between the metal and the semimetal layer and (2) the MR in the transverse Hall configuration for low and intermediate magnitudes of the applied magnetic field.

High-mobility semimetals could also be employed as novel materials for high-speed devices. Due to the mobility and drift velocity of charge carriers higher than those of typical III–V semiconductors and, for most of them, one-two orders of magnitude less than that of charge carriers in semiconductor heterostructures such as AlGaAs/GaAs (measured charge carrier mobility $\mu =35\times 10^{6}$ cm${}^{2}$/(Vs) at *T* close to T=0 K) [166] characterized by the highest measured mobility, a time response either faster or slower with respect to that exhibited by high-speed semiconductor heterostructure devices [167] could be expected. In all these applications, a good choice would be to employ Weyl semimetals, which, in general, exhibit higher charge carrier mobilities if compared with those of Dirac semimetals.

Being inspired by the recent results obtained on the Bi${}_{2}$Te${}_{3}$ nanostructured topological insulator, which exhibits unique nonlinear optical properties under the effect of a laser source in the form of a two-wave mixing [168], some representative topological semimetals could be applied as structured light systems such as optoelectronic or photodetector devices by virtue of their similar topological properties.

Finally, the profound implications of high-mobility semimetal features on the observed MR and on the 3D quantum Hall effects, together with the consolidated knowledge of the well-known magnetotransport properties of semiconductors and graphene, could potentially open the way for novel fields of investigations in materials science.

## Data Availability

No new data were created or analyzed in this study. Data sharing is not applicable to this article.

## Abbreviations

The following abbreviations are used in this manuscript:

AMR	Anisotropic magnetoresistance
BZ	Brillouin Zone
CMR	Colossal magnetoresistance
DFT	Density functional theory
EMR	Extraordinary magnetoresistance
GMR	Giant magnetoresistance
MR	Magnetoresistance
MTE	Magneto-thermoelectric
TMR	Tunnel magnetoresistance
